# microRNA‐30a attenuates TGF‐β1–induced activation of pulmonary fibroblast cell by targeting FAP‐α

**DOI:** 10.1111/jcmm.15020

**Published:** 2020-01-28

**Authors:** Geting Wu, Bin Xie, Can Lu, Chen Chen, Jianhua Zhou, Zhenghao Deng

**Affiliations:** ^1^ Department of Pathology Xiangya Hospital Central South University Changsha China; ^2^ Department of Pathology School of Basic Medicine Central South University Changsha China

**Keywords:** FAP‐α, miR‐30a, MRC5, pulmonary fibrosis, TGF‐β1

## Abstract

Idiopathic interstitial pulmonary fibrosis is a common diffuse interstitial lung disease and has poor prognosis. And one of the pathological features of it is persistent fibroblast activation. It was reported that microRNA‐30a was down‐regulated in bronchoalveolar lavage fluid from idiopathic pulmonary fibrosis patients. But whether miR‐30a is involved in fibroblast activation and its specific mechanism is unclear. In this study, we aimed to investigate the role of miR‐30a in fibroblast activation induced by TGF‐β1. We found miR‐30a could targetedly suppress FAP‐α expression. In MRC5 cells, miR‐30a was not only involved in regulating the expression of FAP‐α, col1a and α‐SMA induced by TGF‐β1 but also had a role in cell proliferation with or without TGF‐β1 treatment via regulating FAP‐α expression. Thus, the results indicated that miR‐30a alleviated fibroblast activation by regulating the expression of FAP‐α.

## 1 INTRODUCTION

Idiopathic pulmonary fibrosis is a common diffuse interstitial lung disease with high mortality and poor prognosis.^1^ Nowadays, the dominant theory explaining pathogenesis of pulmonary fibrosis is that repeated damage leads to increased cell death, impaired re‐epithelialization, and excessive collagen and matrix production caused by persistent fibroblasts activation,^2^ whereas the specific molecular mechanism is still uncertain.

MicroRNAs play crucial roles in pathogenesis of lung fibrosis.^3^ Tu reported that miR‐30 inhibited carbon tetrachloride‐induced liver fibrosis by attenuating TGF‐β1 signalling.^4^It was reported that microRNA‐30a was down‐regulated in bronchoalveolar lavage fluid from idiopathic pulmonary fibrosis patients.^5^ However, the biological function and underlying mechanisms of miR‐30a in pulmonary fibrosis remain largely unclear.

Fibroblast activation protein α (FAP‐α) is considered as a marker of activated fibroblasts. As an inducible cell surface glycoprotein, FAP‐α is a kind of serine protease with post‐proline dipeptidyl peptidase and endopeptidase enzymatic activity.^6^ Existing extensive literature did not only relate to the role of FAP in tumour growing and cancer cell invasion into the ECM 7 but also reported the function of FAP in a number of chronic inflammatory disorders with fibrotic evolution, such as rheumatoid arthritis, cirrhosis and Crohn's disease.^8^, ^9^ Unfortunately, its exact role in fibroblast activation remains largely unknown.

Here, we found that miR‐30a could inhibit fibroblast activation via targeted inhibition of FAP‐α expression. Our results provide convincing evidence that miR‐30a participates in development of pulmonary fibrosis and may be a therapeutic target of inhibition, the development of pulmonary fibrosis.

## 2 METHODS

See supporting information for detailed description.

## 3 RESULTS

### 3.1 TGF‐β1 induces FAP‐α expression in MRC5 cell

In the present study, the expression of miR‐30a was significantly lower in the pulmonary fibrosis model than that in the control group and the expression of FAP‐α mRNA and protein in the lung tissue of the pulmonary fibrosis group was significantly increased compared with the control group (Supp. 1).

**Figure 1:**
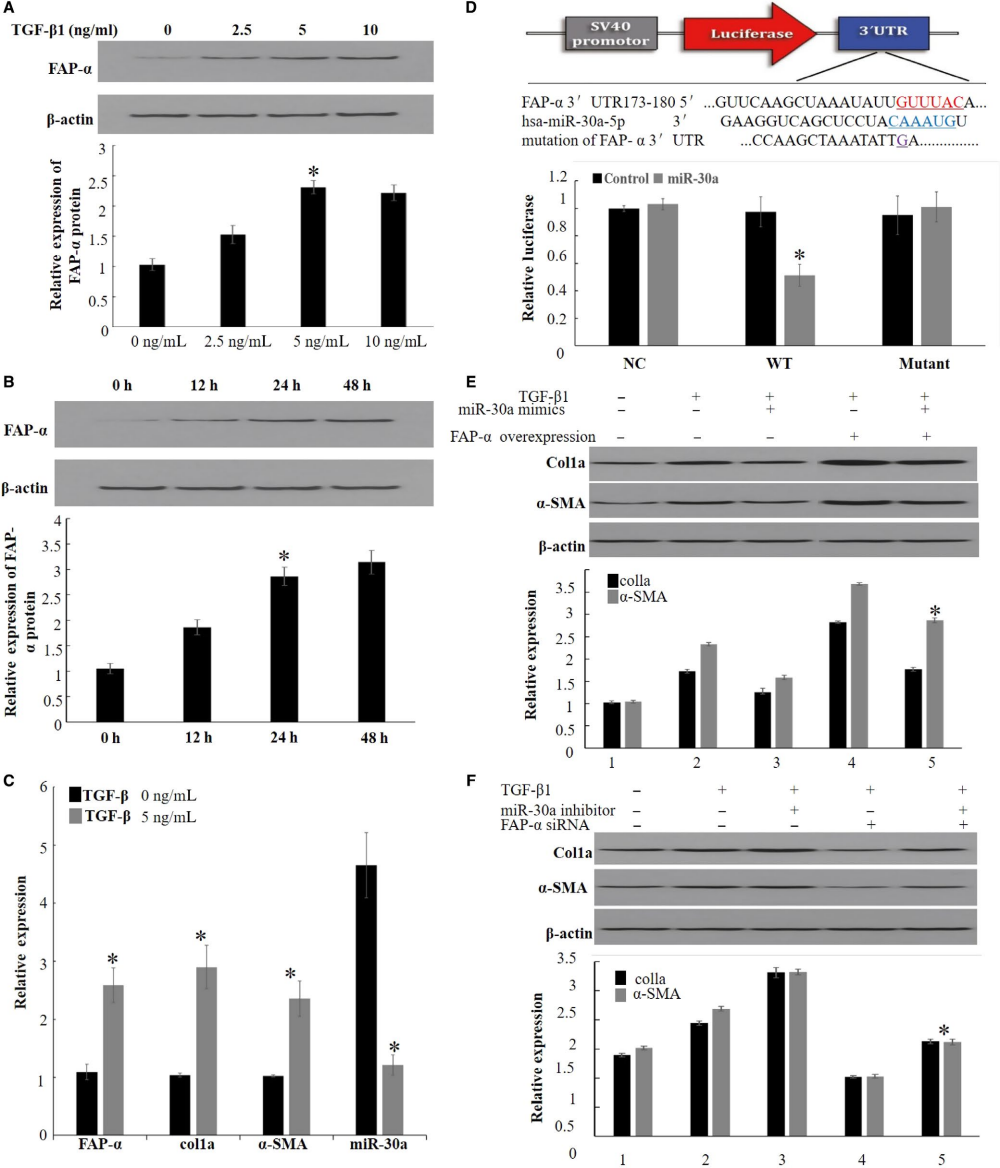
TGF‐β1 increases FAP‐α expression in MRC cell. Effect of TGF‐β1 on the expression of FAP‐α protein was detected by Western blot in MRC cells (A) dosage course. (B) time course. (C).TGF‐β1 changed expression of FAP‐α, col1a, α‐SMA and miR‐30a was detected by qRT‐PCR in MRC cells treated with 5 ng/mL for 24 h. The expression fold changes in TGF‐β1–treated cells were compared with that control group. (D). Based on TargetScan (http://www.targetscan.org/), conserved miR‐30a binding site in the 3’UTR of FAP‐α mRNA was constructed into pmirGLO dual‐luciferase miRNA target expression vector. Luciferase activity was analysed in the MRC cells. MRC cells were cotransfected with miR‐30 mimics, luciferase reporter. (E) miR‐30a mimics attenuated expression of col1a and α‐SMA protein induced by FAP‐α overexpression when co‐treated with TGF‐β1. (F) FAP‐α knockdown attenuated expression of col1a and α‐SMA protein induced by miR‐30a antagomir when co‐treated with TGF‐β1. Data are the means ± SEM of three independent experiments. *,**P < .05

To investigate the effect of TGF‐β1 on expression of FAP‐α, MRC5 cells were treated with TGF‐β1 with dosage and time course. Western blotting data showed that FAP‐α expression was increased in a dosage and time‐dependent manner (Figure 1A,B). When MRC5 cell was treated with TGF‐β1 5ng/mL for 24 h, qRT‐PCR data showed that expression of col1a and α‐SMA was increased obviously, whereas expression of miR‐30a was reduced (Figure 1C). Since expression of col1a and α‐SMA was a marker of fibroblast activation, our results indicated that TGF‐β1–induced MRC5 cell activation accompanied by up‐regulation of FAP‐α expression and down‐regulation of miR‐30a.

### 3.2 FAP‐α is the target of miR‐30a

Bioinformatics analysis revealed miR‐30a could bind to the 3'UTR of the mRNA of FAP‐α. To investigate whether miR‐30a inhibits the expression of FAP—by suppressing the translation of FAP‐α, the 3'UTR of FAP‐α fragment containing the normal or mutant binding site of miR‐30a was cloned into the pmirGLO dual‐luciferase miRNA target expression vector. The results showed that up‐regulation of miR‐30a significantly attenuated the luciferase activity of the wild‐type FAP‐α3'UTR reporter gene, but did not alter the luciferase activity of the mutant reporter gene (Figure 1D). MRC5 cells were then cotransfected with miR‐30 mimics and FAP‐α cDNA, and the results showed that up‐regulation of FAP‐α reversed the expression of col1a and α‐SMA induced by up‐regulation of miR‐30 (Figure 1E). When MRC5 cells were cotransfected with miR‐30 inhibitor and FAP‐α siRNA, it was found that down‐regulation of FAP‐α inhibited the expression of col1a and α‐SMA induced by decreased miR‐30(Figure 1F). This indicated that FAP‐α was a direct target of miR‐30a.

### 3.3 miR‐30a plays an important role in MRC5 cell activation induced by TGF‐β1

**Figure 2:**
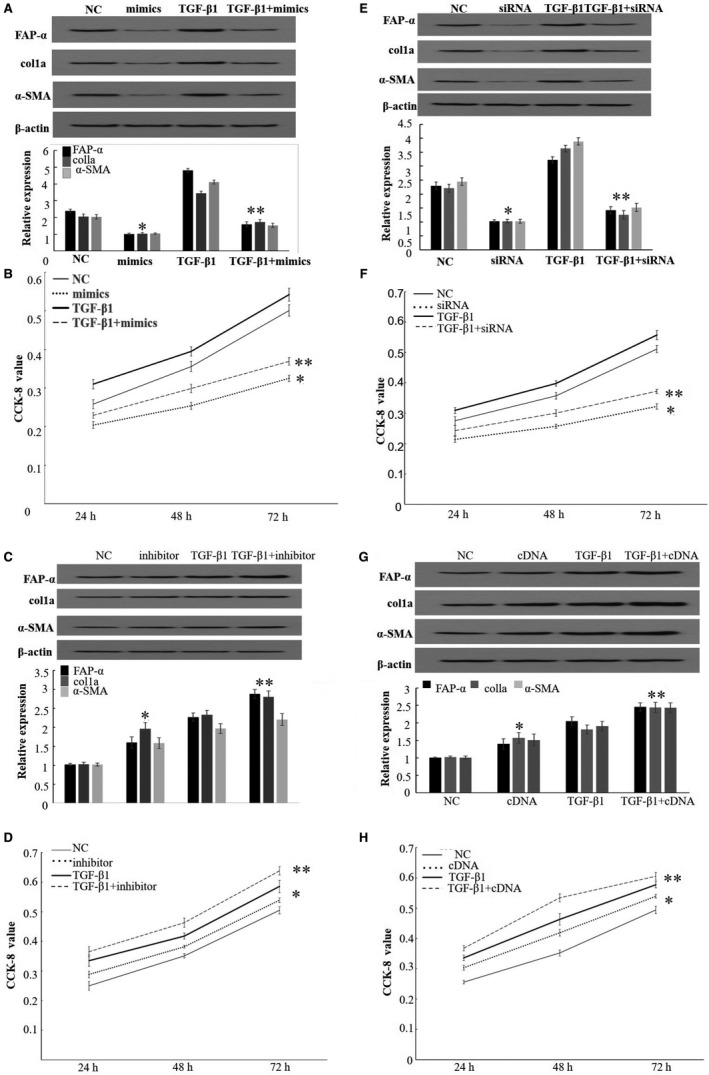
miR‐30a and FAP‐α were involved in MRC cell activation induced by TGF‐β1. (A) miR‐30a mimics decreased expression of FAP‐α, col1a and α‐SMA protein induced by TGF‐β1 in MRC cell which was detected by Western blot. (B) miR‐30a mimics decreased cell proliferation induced by TGF‐β1 in MRC cell which was detected by CCK‐8 assay. (C) miR‐30a inhibitor increased expression of FAP‐α, col1a and α‐SMA protein induced by TGF‐β1 in MRC cell which was detected by Western blot. (D) miR‐30a inhibitor increased cell proliferation induced by TGF‐β1 in MRC cell which was detected by CCK‐8 assay. (E) FAP‐α knockdown decreased expression of FAP‐α, col1a and α‐SMA protein induced by TGF‐β1 in MRC cell which was detected by Western blot. (F) FAP‐α knockdown decreased cell proliferation induced by TGF‐β1 in MRC cell which was detected by CCK‐8 assay. (G) FAP‐α overexpression increased expression of FAP‐α, col1a and α‐SMA protein induced by TGF‐β1 in MRC cell which was detected by Western blot. (H) FAP‐α overexpression increased cell proliferation induced by TGF‐β1 in MRC cell which was detected by CCK‐8 assay. Data are the means ± SEM of three independent experiments. *,**P < .05

To explore the role of miR‐30a in fibroblast activation induced by TGF‐β1, miR‐30a mimics were transfected into MRC5 cell with or without TGF‐β1 treatment. Western blotting data showed that miR‐30a mimics decreased the expression of FAP‐α, col1a and α‐SMA in MRC5 cells. They also decreased the expression of FAP‐α, col1a and α‐SMA induced by TGF‐β1 in MRC5 cells. CCK‐8 assay showed that up‐regulated miR‐30a could inhibit MRC5 cell proliferation with and without TGF‐β1 treatment. (Figure 2A,B).

Then, miR‐30a inhibitor was transfected into MRC5 cell with or without TGF‐β1 treatment. Western blotting discovered that down‐regulated miR‐30a promoted the expression of FAP‐α, col1a and α‐SMA in MRC5 cells with or without TGF‐β1 treatment. Besides, decreased miR‐30a accelerated MRC5 cell proliferation (Figure 2C,D) with and without TGF‐β1 treatment. Those data indicated that miR‐30a had an important role in fibroblast activation induced by TGF‐β1.

### 3.4 FAP‐α is involved in MRC5 cell activation induced by TGF‐β1

To investigate the role of FAP‐α in fibroblast activation induced by TGF‐β1, FAP‐α siRNA was transfected into MRC5 cell with or without TGF‐β1 treatment to inhibit the expression of FAP‐α. FAP‐α siRNA reduced the expression of col1a and α‐SMA in MRC5 cells, as well as in MRC5 cells induced by TGF‐β1. What is more, FAP‐α suppression inhibited MRC5 cell proliferation with and without TGF‐β1 treatment (Figure 2E,F).

However, when FAP‐α was up‐regulated in MRC5 cells with or without TGF‐β1‐treated, it was found to promote the expression of col1a and α‐SMA in MRC5 cells with or without TGF‐β1–treated, accompanied by an increasing ability of proliferation (Figure 2G,H). These data indicated that FAP‐α was involved in TGF‐β1–induced fibroblast activation.

## 4 DISCUSSION

Currently, idiopathic pulmonary fibrosis is considered to be a life‐threatening disease. It is characterized by progressive deposition of collagen and other extracellular matrix (ECM) molecules. Fibroblast activation is an important part of fibrosis development, but its main mechanism is still not very clear.^2^ MicroRNAs are involved in all aspects of pulmonary fibrosis.^10^However, whether miR‐30a is involved in fibroblast activation has not been clarified yet. In the present study, we identified FAP‐α is a target of miR‐30a and demonstrated that miR‐30a could repress TGF‐β1–induced fibroblast activation by down‐regulating the expression of FAP‐α.

MicroRNA‐30a plays an important role in the development of various disease.^3^ Roderburg discovered that miR‐30 family members were abundantly expressed in adult liver tissue and significantly down‐regulated in the process of liver fibrosis.^11^ In this study, we found miR‐30a was involved in regulation expression of FAP‐α, col1a and α‐SMA induced by TGF‐β1 and also promoted the proliferation of MRC5 cells, whereas FAP‐α was a direct target of miR‐30a. Finally, we found miR‐30a inhibited the activation of MRC5 cells by down‐regulating FAP‐α expression.

FAP is significantly up‐regulated in a variety of diseases including liver fibrosis, atherosclerosis and arthritis, and it has gradually become a unique therapeutic target of fibrosis.^12^ In pulmonary fibrosis, Fan et al found that FAP could participate in collagen catabolism and clearance and has a protective effect on pulmonary fibrosis.^13^ Egger et al reported that FAP was overexpressed in the lungs of BLM‐treated mice, whereas the FAP inhibitor PT100 had anti‐fibrogenic effects.^14^ At the same time, Kimura discovered that loss of FAP expression in FAP^+^ cells accelerated fibrosis in the bleomycin model.^15^ In the present study, we found that TGF‐β1 promoted FAP‐α expression in MRC5 cells with the increasing in ability of proliferation, suggesting that it had a potential role in MRC5 activation. Therefore, we provide new evidence that FAP‐α promotes fibroblast activation.

In conclusion, the results of the present study indicated that miR‐30a alleviates fibroblast activation by suppressing the expression of FAP‐α. Those results may deeper our understanding of lung fibrosis and develop novel treatments.

## CONFLICT OF INTEREST

The authors declare no conflict interests.

## AUTHOR CONTRIBUTION

Geting Wu contributed to cell culture and manuscript writing. Bin Xie contributed to gene transfection and Western blotting. Can Lu contributed to statistical tests. Chen Chen contributed to RT‐PCR. Jianhua Zhou corrected the manuscript. Zhenghao Deng designed the experiment and wrote the manuscript.

## Data Availability

The data used to support the findings of this study are available from the corresponding author upon request.
