# Supplementary material for: microRNA‐30a attenuates TGF‐β1–induced activation of pulmonary fibroblast cell by targeting FAP‐α
Source: J Cell Mol Med. 2020 Jan 28;24(6):3745–50. doi: 10.1111/jcmm.15020 (PMC7131934; doi:10.1111/jcmm.15020)
Supplement: Supplementary file 1 [file JCMM-24-3745-s001.docx]

**2.1 Reagents**

Bleomycin was purchased from Nippon Kayaku. MiRNeasy Mini Kit was purchased from QIAGEN. MiRNA Q-PCR Detection Kit was purchased from GeneCopoeia. RevertAid H Minus First Strand cDNA Synthesis Kit and SYBR Green master mixture were purchased from Fermentas. Primers were provided by Sangon Biotech. FAP-α, col1a, α-SMA, β-actin antibody were purchased from Abcam. miR-30a mimics and inhibitor were synthesized by Genepharma. FAP-α siRNA and lentivirus vector carrying human FAP-α gene were purchased from Genepharma. Lipofectamine 2000 was purchased from Thermofisher. CCK-8 kit was purchased from Beyotime. The Masson staining reagent kit was purchased from Zhuhai Beisuo Biotechnology. The immunohistochemistry reagent kit was purchased from Beijing ZSGB-BIO. MicroRNA in situ hybridization kit was purchased from Boster. Other common reagents were all provided by Beijing Dingguo Biotechnology.

**2.2 Animal Model**

Ten clean-grade male C57/BL mice with body weights of 30±2 g were purchased from Hunan SJA Laboratory Animal Co, Ltd. This study was approved by the Ethics Committee of Experimental Animals of Central South University. The entire experimental process strictly followed the "Regulation on the Administration of Laboratory Animals" to reduce pain and increase the comfort of the mice.The mice were randomly divided into the control group and the model group (n=5). According to the method reported by Meng[20], the model of pulmonary fibrosis was established through intratracheally instillation with bleomycin (5 mg/kg) while control group was provided the same dose of saline. On the 28^th^ day, mice were anesthetized with amobarbital (40 mg/kg, intravenously). The left lung tissues were harvested for hemotoxylin and eosin staining and Massons trichrome staining. The right lung tissues were preserved by liquid nitrogen for Real Time-PCR and Western blotting.

**2.3 HE** **and Masson staining**

Lung tissue was fixed in 10% formalin for 24 hrs and conventionally dehydrated, embedded as tissue paraffin blocks. Then it was cut into sections for following experiments at a thickness of 3 µm. Sections were stained by conventional hematoxylin-eosin and Masson trichrome. According to the method reported by Song[21], the areas of inflammatory cell infiltration in HE-stained sections were observed under a microscope to determine the degrees of alveolar inflammation: normal was 0 points, ≤20% was 1 point, >20% but ≤50% was 2 points, and >50% was 3 points. The positive areas in Masson-stained sections were observed under a microscope to determine the degrees of lung fibrosis: normal was 0 points, ≤20% was 1 point, >20% but ≤50% was 2 points, and >50% was 3 points.

**2.4** **RNA isolation and Quantitative Real-time PCR**

As previously described[22],Tissues or cells total RNA were isolated and extracted with TRIzol reagent. miRNAs-specifc stem-loop primer or oligo dT primers was used to synthesize cDNA using reverse transcriptase. The primer against miR-30a was 5'-TGTAAACATCCTCGACTGGAAG3' (forward) and 5'-TGGTGTCGTGGAGTCG-3' (reverse).The primer against FAP-α was 5'-AATGTGGCATAGCAGTGGCT-3' (forward) and 5'-TGTTGGGAGGCCCATGAATC-3' (reverse). The primer against col1a was 5'- AGTGGTTTGGATGGTGCCAA-3' (forward) and 5'- GCACCATCATTTCCACGAGC-3' (reverse).The primer against a-SMA was 5'--ACTGCCTTGGTGTGTGACAA3' (forward) and 5'-TCCCAGTTGGTGATGATGCC-3' (reverse).The primer against GAPDH was 5'-AACTTTGGCATTGTGGAAGG-3' (forward) and 5'-GGATGCAGGGATGATGTTCT-3' (reverse).The primer against U6 was 5'-GCTTCGGCAGCACATATACTAAAAT-3' (forward) and 5'-CGCTTCACGAATTTGCGTGTCAT-3' (reverse) qRT-PCR was carried out on the ABI 7500 Real-Time PCR system (Applied Biosystems, Foster City, CA, USA) . The relative expression of miR-30a was normalized to small nuclear RNA U6, and the expression levels of genes were normalized against GAPDH.

**2.5 Western blotting**

As previously described[23],Tissues or cells were homogenized on ice and added the appropriate

amount of protein lysis buffer. Tissues were centrifuged at 4℃, and the supernatant was collected. The bicinchoninic acid (BCA) reagent kit was uesd to measure the protein concentration. Discontinuous SDS-PAGEA was performed with total of 20 µg of protein. Proteins were transferred onto polyvinylidene fluoride (PVDF) membrane using the semi-dry method. The membrane was blocked in 3% BSA for 2 h and incubated with the rabbit anti-FAP-α,anti-col1a, anti-α-SMA (1:1000) , anti-β-actin antibody (1:5000) at 4℃ for overnight and then the membrane was incubated with the horseradish peroxidase-labeled secondary antibody at room temperature for 2 h. The results were showed using ECL reagent. The relative expressions of FAP-α, col1a and α-SMA were normalized against β-actin.

**2.6 Immunohistochemistry**

The streptavidin peroxidase based immunohistochemistry was performed as previously described[24]. Sections were deparaffinized and rehydrated and autoclave heating was performed to retrieval antigen. 3% hydrogen peroxide was used to block endogenous peroxidases. After washing with PBS 3 times, sections were incubated with primary antibody (anti-FAP-α; 1:500) and incubated at 4℃overnight. Sections were washed with PBS 3 times, and the secondary antibody was applied and incubated at room temperature for 1 h. After sections were washed with PBS 3 times, the streptavidin peroxidase substrate was added and incubated at 37℃for 1 h. Sections were then washed with PBS 3 times, and the results were developed using DAB.

**2.7** **MicroRNA in situ hybridization**

As previously described[25], Digoxin labeled miR-30a-5p probe was obtained from Boster. Sections were cut from paraffin embedded tissues. In situ hybridization was performed with Digoxin labeled miR-30a-5p probe according to manufacturer’s instructions.

**2.8 Cell culture and treatment**

MRC5, the human embryonic pulmonary fibroblast cell line, was cultured in DMEM supplemented with 10% fetal calf serum at 37 °C in a humidified atmosphere of 5% CO2. In the case of TGF-β1 treatment, recombinant human TGF-β1 was added as previously described[26]. MRC5 cells were transfected with FAP-α siRNA or FAP-α cDNA using lipofectamine 2000 at a primary density of 2×105/ml. 50 nM miR-30a-5p mimic or 100 nM miR-30a-5p inhibitor was transfected into MRC5 cells using lipofectamine 2000. Negative sequences were used with the same concentration as controls. After 6 h culture with transfection, the cells were replaced with fresh DMEM medium with antibiotics. All experiments were repeated in triplicate.

**2.9 Cell proliferation assay**

MRC5 cells were breed at primary number of 2,000 cells per well triplicate on 96-well plate.

When grown to approximately 70% confluence, cell was transfected with different materials. After the transfection, cells were incubated for 24h,48h and 72h with or without TGF-β1 stimulation. According to the manufacturer’s protocol, cell counting kit-8 (CCK-8) was used to determine the cell proliferation.

# 2.10 Wound-healing scratch assay

As previously described[27], MRC5 cells were breed with 50,000 cells/cm2 in

6-well plates. Cells were scratched with a 2-μl pipette tip when the cells formed a confluent monolayer and then cells were washed with PBS to remove cell debris. The scratch was visualized using microscopy and photographed, and the width of the wound was measured at 0h, 24h and 48 h.

**2.11 Luciferase reporter assay**

As previously described[28], the wild and mutant type 3’UTR fragments of human FAP-α, were inserted into downstream of the firefly luciferase coding region in pmirGLO dual-Luciferase miRNA target expression vector. Vectors were cotransfected with miRNA or control mimics into MRC5 cells in a 24-well plate. Luciferase activities were measured 48 h after transfection with a bioluminescence plate reader. The activity of firefly luciferase was normalized (firefly luciferase activity / Renilla luciferase activity).

**2.12 Statistical analyses**

Analyses of experimental data were performed using SPSS 17.0 software. Measurement data are expressed as means±standard deviations (x±s). The examination level was bilateral α=0.05. p<0.05 indicated that the difference had statistical significance.
